# Acute graft versus host disease after orthotopic liver transplantation

**DOI:** 10.1186/1756-8722-5-50

**Published:** 2012-08-13

**Authors:** Inga Mandac Rogulj, Joachim Deeg, Stephanie J Lee

**Affiliations:** 1 University of Zagreb School of Medicine, University Hospital Merkur, Zagreb, Croatia; 2 Clinical Research Division, Fred Hutchinson Cancer Research Center and Department of Medicine, University of Washington, Seattle, USA

## Abstract

Graft versus host disease (GVHD) is an uncommon complication after orthotopic liver transplantation (OLT) with an incidence of 0.1–2%, but an 80–100% mortality rate. Patients can present with skin rashes, diarrhea, and bone marrow aplasia between two to eight weeks after OLT. Diagnosis of GVHD is made based on clinical and histologic evidence, supported by chimerism studies showing donor HLA alleles in the recipient bone marrow or blood. Several therapeutic approaches have been used for the management of GVHD after OLT including increased immunosuppression, decreased immunosuppression, and cellular therapies. However, success rates have been low, and new approaches are needed.

## Introduction

The first successful human orthotopic liver transplant (OLT) was performed in 1967 by a surgical team led by Dr. Thomas Starzl in Denver, Colorado. Today more than 6000 liver transplants are performed each year in the United States with a one year survival rate of 80–85% [1].

Acute graft versus host disease (GVHD), a frequent complication after hematopoietic cell transplantation (HCT), occurs infrequently after solid organ transplantation [2]. However, GVHD has been reported following blood transfusions [3], and any procedure that transfers viable allogeneic lymphocytes into another individual carries the potential risk of inducing GVHD. Thus, among solid organ transplants GVHD is more likely in recipients of liver or intestinal transplants where the transplanted organs contain high numbers of donor lymphocytes [2]. After OLT the incidence of GVHD has been estimated at 0.1–2%, but the mortality rate is 80–100% [4,5]. Those estimates are derived from single institution studies; the United Network for Organ Sharing (UNOS) in the United States does not routinely collect information about GVHD, and the absence of registry studies from other parts of the world suggests that this complication is not routinely captured in databases. Generally the same treatment principles are applied as with GVHD after HCT. However, the likelihood of success is low, and new approaches are needed.

## Case presentation

A 53-year-old woman had a 14 year history of primary biliary cirrhosis and received a liver transplant from a cadaveric male donor. Transplantation was uncomplicated and she received 3 doses of rabbit antithymocyte globulin (ATG) induction peritransplant. Post-transplant immunosuppression was with tacrolimus and mycophenolate mofetil. She was discharged from the hospital on post-operative day (POD) 14. Prednisone 5 mg daily was added on POD 20 for additional immunosuppression. On POD 38 she was readmitted with tremors, forgetfulness, headache and difficulty with word finding. Her absolute neutrophil count was 0.09 × 10^9^/L. A bone marrow biopsy showed a markedly hypocellular marrow with no evidence of dysplasia. Flow cytometry showed less than 0.1% CD34 positive progenitors consistent with severe myelosuppression. Bone marrow chimerism studies showed 80% liver donor and 20% host cells. Cytogenetic analysis was limited to one metaphase, which showed a 46 XY karyotype, consistent with male donor origin. Due to the pancytopenia and documented donor chimerism, the working diagnosis was GVHD restricted to the bone marrow. She had no nausea, diarrhea or skin rash suggestive of gastrointestinal or skin GVHD, and liver GVHD would not be expected given the OLT. She was treated with rabbit anti-thymocyte globulin (ATG) 1.5 mg per kg for three consecutive days and alefacept 30 mg per dose, twice weekly for three doses. Tacrolimus, mycophenolate mofetil and prednisone were discontinued. HLA-typing of her two siblings was initiated.

Follow-up bone marrow biopsy 12 days after administration of ATG showed continued aplasia with < 0.1% CD34+ cells. Bone marrow chimerism was 27% donor and 73% host, while unsorted peripheral blood chimerism showed 98% donor and 2% host cells. No further bone marrow studies were done.

On POD 53 after OLT and day 7 after starting ATG, she developed an erythematous, pruritic and painful skin rash, most prominent on her hands but also extensively involving her back, arms and legs. Skin biopsy was consistent with GVHD, and chimerism studies showed male (donor) lymphocytes in the biopsy specimen. She was treated with topical steroids for these lesions given the high risk of infection and the fact that the lesions were not progressing.

Her subsequent clinical course was complicated by prolonged vancomycin-resistant enterococcal bacteremia, which was treated with multiple antibiotics and granulocyte transfusions. Weekly rituximab was administered for EBV viremia and abdominal CT showing retroperitoneal lymphadenopathy, suggestive of post-transplant lymphoproliferative disorder.

Liver function tests were normal throughout her course. HLA-typing showed that the liver donor shared a single DQB1 antigen with the patient, but the donor was heterozygous at all loci. Thus, the HLA typing of donor and patient would not have predicted a high risk for graft-versus-host disease.

Due to the profound bone marrow failure, the plan was to proceed with allogeneic HCT. However, the patient’s clinical condition deteriorated rapidly, and she died POD 86 from septicemia, pulmonary aspergillosis and respiratory distress syndrome before a donor was identified.

## The spectrum of clinical presentation

In 1988, Burdick and colleagues first described acute GVHD after OLT, and more than 80 cases have been reported since then [6]. Graft-versus-host disease after OLT usually presents at two to eight weeks postoperatively with bone marrow aplasia, skin rash and diarrhea. Liver function is not affected because the transplanted liver lacks host antigen. The diagnosis may be delayed, because similar presentations can be seen with drug reactions and bacterial or viral infections. One of the earliest clinical presentations is an erythematous maculopapular skin rash. Detection of donor lymphocytes in skin and a nonspecific lichenoid reaction pattern with basal vacuolar changes, dyskeratosis and apoptotic cells in the epidermis may be seen [7]. Donor cells are located at the dermoepidermal junction and migrate upward into the epidermis; FISH for the Y chromosome may confirm donor cells when donor and recipient are sex mismatched [8,9].

Diarrhea is most commonly the result of absorptive function loss caused by lymphocyte infiltration and destruction of the intestinal mucosa, and is more evident as the post-transplant period lengthens. Biopsy specimens of gastric, duodenal or colonic mucosa demonstrate apoptosis and infiltration by donor lymphocytes [10]. In case of pancytopenia, and suspected GVHD restricted to the bone marrow, a bone marrow analysis usually shows a markedly hypocellular marrow with no evidence of dysplasia. Flow cytometry is consistent with severe myelosuppression. Bone marrow chimerism studies show a high percentage of liver donor and significantly less host cells. Cytogenetic analysis is consistent with donor karyotype. Most patients die from infections, bone marrow failure, gastrointestinal bleeding or multiorgan failure. As with transfusion-associated GVHD, the high mortality rate after OLT may explain why no clearly documented cases of chronic GVHD have been reported.

## Pathophysiology and diagnosis of GVHD

In OLT, about 10^9^–10^10^ donor-derived leukocytes including monocytes, T-cells and natural killer cells are transplanted with the liver allograft [11], approximately the same number as with a standard peripheral blood stem cell infusion. This infusion of immunocompetent cells and pluripotent progenitors, in conjunction with the immunosuppression used for the liver transplant, resembles the circumstances of an immunosuppressed patient receiving a hematopoietic stem cell transplant. In fact, donor lymphocytes can be routinely found within three weeks and even up to 100 days after OLT in the peripheral blood and bone marrow [12,13], and detectable chimerism is associated with a decreased risk of liver rejection. In most cases, over time the immune system of the patient rejects the donor’s lymphocytes and full host chimerism is re-established. Lymphocyte populations in the donor liver are distinct from those in peripheral blood with a reversed ratio of CD4:CD8 among hepatic lymphocytes and a higher proportion of CD8*+* cells and B lymphocytes [14]. Lymphocytes transferred with the donor’s liver may induce severe pancytopenia by attacking and destroying the recipient’s hematopoietic stem cells. Donor stem cells with pluripotent function can migrate from the liver to the recipient’s bone marrow, spleen, lymph nodes and lungs and give rise to mature donor cells of several hematopoietic lineages [15]. However, the number of hematopoietic stem cells transplanted with the donor liver is not sufficient and the degree of HLA mismatching is too great to allow engraftment and reconstitution of the myeloid and erythroid lineages. Recipients with GVHD can experience symptoms of upper and lower gastrointestinal involvement with nausea, vomiting, and diarrhea, and skin involvement with maculo-papular rashes and erythema. These clinical signs and symptoms are identical to what is seen in primary hematopoietic cell transplantation.

Engraftment of donor lymphocytes can be confirmed by chimerism studies and identification of donor HLA alleles in the recipient bone marrow or blood using a polymerase chain reaction (PCR) test [16]. A DNA sample from the liver donor is required to confirm the source of allogeneic cells. By convention the diagnosis of donor engraftment is confirmed if the donor T-cell level is >1% of T-cells detected. Persistently elevated levels of donor CD8 + T/NK cells in the periphery (>10%) may be indicative of GVHD. The level of donor CD8+ T and NK cells may correlate with the severity of GVHD manifestations [17]. Early recognition and expeditious treatment for GVHD is important to control the immune reaction before it is too established and extensive organ damage has occurred.

Domiati-Saad and colleagues reported serial single tandem repeat results in 49 patients during the first two months after OLT. In 38 of the 49 patients, donor T cell chimerism was present on POD 2, and remained positive by the end of the first week in 23 patients. Donor CD8+ T-cell chimerism was less than CD3+ T cell chimerism measured on the same sample, but in the two patients with acute GVHD the level of CD8+ T-cell chimerism was generally equal to or greater than the CD3+ T-cell chimerism. These two patients had characteristic signs of acute GVHD. Donor CD3+ fractions above 10–20% of lymphocytes correlated strongly with acute GVHD [18].

## Risk factors for GVHD

Shared HLA antigens between donor and recipient are the most important risk factors for the induction of GVHD. Use of an HLA-homozygous donor, resulting in one way HLA matching in the GVHD direction, significantly increases the risk of developing GVHD after OLT [19].

Kamei and colleagues investigated the incidence of fatal GVHD following living donor liver transplantation (LDLT) in a retrospective study of 906 donor-recipient pairs. They analyzed the ages of the recipient and donor, donor relationship, original disease, initial symptoms, onset and course of GVHD, Glucksberg stage, and donor/recipient HLA type. Eight cases (1%) of fatal GVHD after LDLT were identified with skin rash as the most common initial manifestation. Seven cases had unidirectional HLA matching at 3 loci (HLA-A, -B and –DR) in the GVHD direction (donor homozygous). Case 8 had unidirectional HLA matching at 2 loci (HLA-A and –DR) in the GVHD direction with the B locus matched because of recipient homozygocity. Among these 8 cases, four donors were children, three were parents and one was a sibling. The authors suggested that the risk of fatal GVHD following LDLT may depend on the number of loci with donor-dominant one-way HLA matching, and was highest with mismatching in the GVHD direction at all 3 loci (HLA-A, -B and –DR) [20].

Key and colleagues reported a single-center analysis of 412 consecutive adult OLT recipients where HLA typing data were available. Their data showed that HLA matching at HLA-B, but not HLA-A or –DR was a significant risk factor for the development of GVHD. Of 14 donor-recipient pairs matched at HLA-B, three (21%) developed GVHD [21].

Chan and colleagues studied 205 OLT patients surviving more than 30 days. Four patients (2%) developed GVHD, and three of them had received a donor liver with steatosis graded mild or more severe. This analysis did not identify HLA-matching as a risk factor for GVHD, and could not confirm cryptogenic cirrhosis (as an indication for liver transplantation) or older recipient age (>40 yrs) to be predictors of GVHD. Alcoholic liver disease, particularly in combination with hepatocellular carcinoma and glucose intolerance, conferred a very high risk for GVHD. They concluded that immunodeficient states due to diabetes mellitus type I and II, various autoimmune diseases and hepatocellular carcinoma increased the risk for developing GVHD [22]. Infection with CMV and HSV viruses can depress immunocompetence and increase the risk for GVHD after OLT [23].

Although GVHD after OLT usually appears two to eight weeks after OLT, similar to what is seen in hematopoietic cell transplantation, Pollack et al reported a case of a 52-year-old liver transplant recipient who developed gastrointestinal symptoms and severe aplasia 8 months after OLT. After elimination of all other possible causes of these symptoms, chimerism analysis was performed and showed that 96% of the peripheral blood mononuclear cells were of liver-donor origin. An allogeneic peripheral blood HCT was carried out, but the patient died of septicemia 5 days after transplantation [24]. Severe aplastic anemia is a known late complication after liver transplantation, sometimes occurring years after the transplant [25-29]. Since chimerism studies were not performed in the majority of these cases, it is possible that GVHD could have been a contributing factor.

## Treatment

Several therapeutic approaches have been proposed in the literature. One strategy has been to decrease immunosuppression, in an effort to shift the balance in favor of recipient immunity, but most investigators have tried to increase immunosuppression.

The literature describes cases where patients were treated with corticosteroids as monotherapy because of their well-known ability to induce apoptosis in lymphocytes and profound anti-inflammatory effect. However, high doses of corticosteroids did not seem to improve response rates and may increase the risk for these patients to die of complications of GVHD, infection and multi-organ failure due to side effects of high doses of corticosteroids.

Treatment with immunosuppression beyond steroids has been very heterogeneous and has included antimetabolites, alkylating agents, anti-T cell antibodies, anti-B cell antibodies, intravenous immunoglobulin, cytokine inhibitors, immunostimulants and cellular therapy, including T cell infusions and hematopoietic stem cell transplantation.

The literature may reflect heavy publication bias, where successful cases are reported, but unsuccessful treatment attempts are not. In cases of GVHD-associated marrow failure, standard treatments for GVHD have not been successful.

The Table 1 summarizes the treatment approaches that have been published, but outcome data are not provided, because many immunosuppressants were used in combination, and no clear pattern of success or failure emerged from reviewing the cases.

**Table 1:** Approaches used in the treatment of acute graft-versus-host disease following orthotopic liver transplantation

**Most common treatments**	**Corticosteroids (most), calcineurin inhibitors (many), granulocyte colony stimulating factors (many)**	
Antimetabolites	Azathioprine [30]	
Alkylating agents	Cyclophosphamide [24]	
Anti-T cell antibodies	Anti-thymocyte globulin [5,19,24,31-45]	
	OKT3 [24,42]	
	Campath [38,46,47]	
	Alefacept [43]	
Anti-B cell antibodies	Rituximab [46,48]	
Immunoglobulin	Immunoglobulin [4,49,50]	
Cytokine inhibitors	Infliximab (TNF) [3,31]	
	Etanercept (TNF) [51]	
	Daclizumab (IL2R) [3,38,41,46,52-54]	
	Basiliximab (IL2R) [5,39,44,47,48,55,56]	
Immunostimulants	Thymosin alpha 1 [4,41]	
Cellular therapy	Ex vivo T cell expansion [47]	
	Hematopoietic cell transplantation [24,32,34]	

Anti-T cell antibodies have shown some success in treating corticosteroid resistant GVHD, but are associated with an increased risk for development of post-transplantation lymphoproliferative disease and viral reactivation. Weekly monitoring by PCR for CMV and EBV reactivation is recommended after starting antilymphocyte therapy.

The second most common approach is to administer cytokine inhibitors, most commonly against IL2 and TNF-α. Anti-interleukin-2 receptor antibodies daclizumab and basiliximab have shown some benefits if introduced early after high-dose corticosteroids. Infliximab is a chimeric mouse/human immunoglobulin-G antibody directed against soluble and transmembrane forms of human TNF-α. Some authors suggest that infliximab should be introduced earlier after the diagnosis of steroid refractory acute GVHD, and if possible, before ATG to avoid increased risks of infection and subsequent lymphoproliferative disorders [31]. Etanercept, a soluble TNF-receptor, has been used successfully at the standard dose of 25 mg twice a week for 8 weeks, although treatment was complicated by invasive Aspergillus infection. The authors recommended antifungal prophylaxis when etanercept is used in acute GVHD because of its significant rate of serious fungal complications [51]. The experience with HCT in cases of OLT-associated GVHD is very limited.

There is one report of a haploidentical transplant for GVHD-associated pancytopenia, with documented transient engraftment of the haploidentical donor cells followed by rejection of the donor graft and recovery of recipient hematopoiesis. The haploidentical donor was related to the recipient (family member) and different from the liver donor [32].

Another report showed successful harvest and ex vivo expansion of recipient T cells, which upon re-infusion were successful in combating GVHD [57]. Thus, allogeneic HCT could be a treatment for OLT-associated GVHD, although the choice of conditioning regimens is difficult in this complicated patient population. Although the risk of GVHD after OLT is low, the high morbidity and mortality of this complication demand efforts to prevent it. Minimizing immunosuppression of the recipient or decreasing the lymphocyte burden in the donor liver might prevent GVHD but increase the rate of liver graft rejection [58]. Depletion of T-lymphocytes from the liver before transplantation could be achieved by treating the cadaveric donor with ATG, but eliminating lymphocytes from the liver may have an adverse effect on graft survival. Stable high donor chimerism has been associated with tolerance to the donor liver [46,59]. Some patients have been able to stop all immunosuppression after OLT; hematopoietic chimerism was not tested in this study but would be interesting to investigate [60]. Avoidance of donors homozygous at HLA loci shared with a patient would lower the risk of GVHD, but this information is not typically available in a timely fashion when the orthotopic liver is being allocated. Avoidance of peritransplant T cell depleting antibodies would help lower GVHD, but might also increase the rate of liver rejection. Conversely, increasing immunosuppression to try to prevent GVHD or introducing agents more commonly used for GVHD prophylaxis in hematopoietic cell transplantation, such as methotrexate, just to prevent a complication seen in <2% of the population may not be warranted, and could actually increase the rate of GVHD if host immunity is more suppressed than donor immunity.

It is clear that more successful approaches to treating OLT GVHD need to be developed. Such advances will require a coordinated effort among transplant centers to systematically test potential treatment algorithms given the rarity of this complication.

## Summary

GVHD after OLT is a rare problem, but one associated with a greater than 80% mortality. Rapid diagnosis, immunosuppressive treatment, and aggressive supportive care are key to potentially reversing this otherwise fatal condition. Our patient presented with symptoms five weeks after OLT, which is similar to previously reported cases. In cases of pancytopenia due to GVHD, rapid evaluation for possible HCT may offer the best option for survival. In conclusion, the diagnosis and treatment of GVHD after solid organ transplantation is difficult because the early symptoms are nonspecific, and no standard treatment is available. Reducing the risk factors for development of GVHD, identifying the disease promptly, preventing infection, and starting treatment as early as possible may improve the dismal outcome associated with this disease. It would be helpful to report cases to registries and world data banks that collect data from organ transplant centers in order to better understand this complication and allow for the design and testing of treatments to try to improve survival.

## Competing interests

The authors declare that they have no competing interests.

## Authors’ contributions

IMR and SJL designed research and wrote the paper, JD revised the paper. All authors read and approved the final manuscript.
